# Serum Procalcitonin Level Is Associated with Positive Blood Cultures, In-hospital Mortality, and Septic Shock in Emergency Department Sepsis Patients

**DOI:** 10.7759/cureus.7812

**Published:** 2020-04-24

**Authors:** Amanda L Webb, Nicholas Kramer, Tej G Stead, Rohan Mangal, David Lebowitz, Larissa Dub, Javier Rosario, Mihir Tak, Srikar Reddy, James R Lee, Jeffrey Adams, Paul R Banerjee, Michelle Wallen, Latha Ganti

**Affiliations:** 1 Emergency Medicine, University of Central Florida College of Medicine, Orlando, USA; 2 Emergency Medicine, Brown University, Providence, USA; 3 Emergency Medicine, Johns Hopkins University, Baltimore, USA; 4 Internal Medicine, University of Central Florida, Orlando, USA; 5 Internal Medicine, University of Central Florida College of Medicine, Orlando, USA; 6 Emergency Medicine, University of Central Florida, Orlando, USA; 7 Emergency Medicine, Osceola Regional Medical Center/University of Central Florida, Kissimmee, USA; 8 Emergency Medicine, Envision Physician Services, Nashville, USA; 9 Emergency Medicine, University of Central Florida College of Medicine/Hospital Corporation of America Graduate Medical Education Consortium of Greater Orlando, Orlando, USA; 10 Emergency Medical Services, Polk County Fire Rescue, Bartow, USA

**Keywords:** sepsis, procalcitonin

## Abstract

This study examines the accuracy of initial and subsequent serum procalcitonin (PCT) levels in predicting positive blood cultures, in-hospital mortality, and development of septic shock in emergency department (ED) patients with severe sepsis. This study includes all patients who presented to our ED with an admission diagnosis of severe sepsis over a period of nine months. The median initial PCT was 0.58 ng/mL, interquartile range (IQR) 0.16-5.39. The median subsequent serum PCT was 2.1 ng/mL, with an IQR of 0.3-11.1. The PCT trend over the initial three hours increased in 67% of the study population. Blood cultures were positive in 38% of the cohort. The median maximum PCT in those with a negative blood culture was 1.06 ng/mL compared to 4.19 ng/mL in those with a positive blood culture (p=0.0116). Serum PCT levels >2.0 ng/mL display significant correlation with positive blood cultures, in-hospital mortality, and development of septic shock and as such may serve as a biomarker for more serious infections.

## Introduction

In the United States, the Center for Disease Control estimates that approximately 1.5 million people develop sepsis annually, of which 250,000 people die [1]. Early recognition of sepsis remains a diagnostic challenge, and this lack of recognition is a large reason for the high rate of morbidity and mortality. In a survey of 1,000 physicians in Europe and the United States, 45% of them claim they may have missed a sepsis diagnosis [2]. Reliable biomarkers that help clinicians identify sepsis early would serve as useful diagnostic tools for early sepsis management. 

Currently, there are many nonspecific markers for diagnosing sepsis, which include: white blood cell count, C-reactive protein (CRP), TNF-alpha, and interleukins. It is important to distinguish the cause of the inflammation (bacterial or viral) and thereby act appropriately in the initial care of the patient. In recent years, procalcitonin (PCT) has shown diagnostic and prognostic value, aiding in antibiotic selection and monitoring of therapy in infectious settings. PCT has shown a high specificity for bacterial causes of inflammation in patients presenting to the Emergency Department (ED) in severe sepsis or septic shock [3]. Rapid elevation in the concentration of PCT and its correlation with the severity of illness make it an ideal biomarker for bacterial infection [4]. Additionally, it has been shown that PCT rises faster than CRP; if the patient adequately responds to treatment, PCT can return to baseline levels faster than CRP, making it a preferred biomarker not only for diagnosis of sepsis but for trending appropriate care and patient improvement. In the present time, a positive blood culture is considered the gold standard for confirmation of bacteremia, but these results take hours to days [5]. Given the urgency of treatment required for management of patients in septic shock, an elevated initial PCT can help confirm an early diagnosis [6]. Although the use of PCT has been gaining prominence as a sepsis biomarker, the Infectious Diseases Society of America has not fully endorsed its use, citing limited and ambiguous evidence.

In this study, the authors sought to determine whether initial serum PCT levels are predictive of positive blood cultures in patients with severe sepsis or septic shock and to add to the growing body of literature regarding PCT’s value in guiding sepsis care.

## Materials and methods

This study included all patients with an admission diagnosis of severe sepsis who presented to our ED during a nine-month period beginning in July 2016. Inclusion criteria were: adult patients over the age of 18 who had an admission diagnosis of severe sepsis, defined as sepsis plus evidence of organ dysfunction, hypoperfusion, or hypotension (systolic blood pressure less than 90 mmHg or a rapid decrease from baseline) as per International Consensus guidelines [7]. Exclusion criteria were: patients who did not present via the ED, and those under the age of 18.

The study was conducted at an academic teaching hospital and level 2 trauma center. This facility has a standardized sepsis laboratory bundle including PCT assay which is repeated within three hours and often again by the inpatient team. The PCT test was performed as an automated test for the determination of PCT in human serum or plasma.

The dataset was developed using a data collection sheet and included demographic, laboratory, and hospital outcome variables. Data were abstracted onto pre-designed data collection sheet and were analyzed with the JMP Pro statistical software, version 14.1 (SAS institute, Cary, NC). A p-value of <0.05 was set for statistical significance. 

The aim of this study was to determine whether PCT levels correlated to any positive blood cultures in this septic cohort. We examined the initial PCT drawn in the ED, the three-hour repeat PCT level, and the difference in the two measurements (the independent variables in the analyses). The outcomes of interest (dependent variables) were: 1) positive blood culture, 2) septic shock (meaning who went on to develop shock, and 3) in-hospital death. Septic shock was defined as persistent hypotension and hypoperfusion despite adequate fluid resuscitation of a 30 cm^3^/kg bolus.

## Results

The cohort comprised 148 patients of which 45% were female. The median age was 72 years, interquartile range (IQR) 60-82 years, and range of 19-98 years. Seventeen percent of patients arrived from a nursing home (NH), and 65% were transported by emergency medical services (EMS).

Procalcitonin was drawn at initial presentation, and repeated at three hours. Initial values, maximum values, and upward versus downward trend were recorded. The median initial PCT was 0.58 ng/mL, IQR 0.16-5.39. The median maximum serum PCT was 2.1 ng/mL, with an IQR of 0.3-11.1. The PCT trend over the initial three hours increased in 67% of the study population. Blood cultures were positive in 38% of the cohort. The median maximum PCT in those with a negative blood culture was 1.06 ng/mL compared to 4.19 ng/mL in those with a positive blood culture (this result is significant with a p-value of 0.0116 using Mood’s median test, a nonparametric test that compares the medians of two independent samples).

We separated patients into two groups based on maximum PCT levels: <2 ng/mL and >=2 ng/mL. For each outcome (blood culture positive, death, readmission within 30 days, type of sepsis, and admission to the ICU) we performed a nominal logistic regression model with age, gender, and PCT group (low or high) as the factors. Odds ratios and p-values are shown in Tables 1-3. Reported p-values are multiplied by five due to the Bonferroni correction for multiple analyses. This is the most conservative statistical correction for multiple analyses. ICU admission and readmission within 30 days were not reported as there were no significant results. Reported p-values are based on the likelihood ratio test, a statistical test which tests the whole model against the model with that factor removed. Thus, a significant p-value means that the model is made significantly worse by excluding that effect. Where a result is written as NS, it means it was not significant.

**Table 1:** Factors predicting in-hospital death. PCT, procalcitonin; CI, confidence interval

Factor	Odds ratio (95% CI)	Adjusted p-value
PCT level	3.67 (1.54-8.70)	0.0100
Age in years	1.056 (1.022-1.091)	0.0015
Gender	NS	NS

From this, we conclude that a PCT level >=2 ng/mL results in 3.67 times increased odds of in-hospital death, and that every increase in age by one year corresponds to 5.6% increased odds of in-hospital death. 

**Table 2:** Factors predicting positive blood culture. PCT, procalcitonin; CI, confidence interval

Factor	Odds ratio (95% CI)	Adjusted p-value
PCT level	2.75 (1.37-5.53)	0.0185
Age	NS	NS
Gender	NS	NS

From this, we conclude that a PCT level >=2 ng/mL results in 2.75 times increased odds of positive blood culture.

**Table 3:** Factors predicting septic shock (as opposed to severe sepsis). PCT, procalcitonin; CI, confidence interval

Factor	Odds ratio (95% CI)	Adjusted p-value
PCT level	3.35 (1.67-6.70)	0.0025
Age	NS	NS
Gender	NS	NS

From this, we conclude that a PCT level >=2 ng/mL results in 3.35 times increased odds of septic shock.

## Discussion

Procalcitonin is an important prognostic indicator in septic patients, with regard to severity of sepsis [8] and in-hospital mortality [9]. In this study, when controlling for other variables, PCT values above 2.0 ng/dL were associated with positive blood cultures, increased in-hospital mortality, and septic shock.

Other studies have suggested the predictive value of PCT as a marker for bacteremia; and the results of this study are in line with this data in the current literature [10]. In a meta-analysis of 16,514 patients with suspected infection, 3420 were diagnosed with bacteremia. It was found that PCT level of 0.5 ng/mL had a sensitivity of 76% and a specificity of 69% for bacteremia [11]. In an observational study of 35,343 consecutive patients undergoing PCT assays and blood cultures for suggested blood stream infection, the negative predictive value for Gram-negative and Gram-positive bacteria was 98.9% and 98.4% respectively for a PCT value of ≥0.4-≤0.75 ng/mL [12].

There are limitations to the application of PCT. PCT can be useful as a biomarker to differentiate between bacterial infection and other pathogenic infection sources. However, PCT levels may be elevated in clinical situations apart from infections including major surgeries, malignancies, severe burns, and chronic kidney disease [13]. This indicates that PCT should not be broadly applied to all patients but is of particular use when selectively applied to settings in which bacterial sepsis is suspected.

Furthermore, many hospital labs do not have capacity for rapid PCT assay. This likely has limited the quantity of studies on this topic and may limit PCT’s usefulness in an emergency setting at some institutions. As we continue to learn more about PCT, its usefulness continues to expand beyond simply identifying or confirming our suspicions of a bacterial infection, but may allow us to start prognosticating for patients, from the time of their ED arrival and throughout their initial stay. This could inform our understanding of who is more likely to survive hospitalization or to require a lengthy hospital stay; and it may change practice about escalation or discontinuation of antibiotics and transition from intensive care to a medical floor.

Further analysis could elucidate more specific applications of PCT, such as a study that attempted to use the value of PCT to differentiate Gram-positive from Gram-negative infections [14]. Moreover, there could be more analysis conducted on the degree of change of PCT as an infection progresses or improves with appropriate antibiotic treatment.

## Conclusions

Elevated serum PCT levels appear to be significantly correlated with positive blood cultures, in-hospital mortality and septic shock, and as such may serve as a biomarker for more serious infections. The results of this study should be interpreted with caution as they reflect the limitations of a small single center study.
